# PERK and IRE1α promote exosome secretion *via* blocking lysosomal degradation of multiple vesicular body

**DOI:** 10.1016/j.jbc.2025.110504

**Published:** 2025-07-21

**Authors:** Shixin Zhou, Zihan Zhou, Si Chen, Ming Wang, Likun Wang

**Affiliations:** 1 State Key Laboratory of Biomacromolecules, Institute of Biophysics, Chinese Academy of Sciences, Beijing, P.R. China; 2 College of Life Sciences, University of Chinese Academy of Sciences, Beijing, P.R. China

**Keywords:** unfolded protein response, extracellular vesicle, lysosome, IRE1α, PERK

## Abstract

The unfolded protein response (UPR) initiated under endoplasmic reticulum (ER) stress can not only maintain the ER homeostasis but also modulate the secretion of proteins and lipids that transmit ER stress signals among cells. Exosomes are multivesicular body (MVB)-derived extracellular vesicles, constituting the unconventional protein secretion pathway. Whether and how the secretion of exosomes is regulated by the UPR remains largely unknown. Here, we reported that ER stress induces exosome secretion in an UPR-dependent way. Activation of PERK and IRE1α, two of the UPR branches, represses the acidification and catabolic activity of lysosomes. This blocked MVB-lysosome fusion, redirecting MVBs from lysosomal degradation to plasma membrane fusion, resulting in exosome release. Calcium-mediated activation of PERK, in the absence of ER stress, is sufficient to suppress lysosomal degradation and augment exosome secretion, partly through its downstream factor ATF4. Our study revealed a function of PERK and IRE1α in modulating lysosome activity and dictating the fate of MVBs, facilitating cell-to-cell communication *via* exosomes.

The endoplasmic reticulum (ER) is the central compartment in eukaryotic cells for protein and lipid processing and transport. As the first destination of many secretory and membrane proteins, it provides a unique environment for protein folding and posttranslational modification (1). However, intrinsic and extrinsic stresses may lead to misfolded protein accumulation in the ER, a condition called ER stress. This happens when proteins are overloaded, folding is unsuccessful, or protein transport is inefficient (2, 3). Abundant in enzymes essential for lipid biosynthesis, the ER also centers in lipid provision, which is crucial for cell’s compartmental organization (4, 5, 6). Both ER stress and disruption of lipid composition in the ER can initiate stress response, the unfolded protein response (UPR), a signal transduction system that originates from three ER transmembrane proteins, that is, IRE1α, PERK, and ATF6 (7, 8, 9, 10). The UPR can rebalance the inputs and outputs of ER proteins, adjust lipid synthesis preference, and reshape organelle interactions so as to maintain the normal ER function or actively initiate programmed cell death (11, 12, 13, 14, 15). Except for these intracellular outcomes, the UPR may also broadcast the ER stress and lipid stress signals among cells. For example, immunoglobulin and cytokine production has been reported to be regulated by the UPR, mediating immune response (16). Different species of lipids, including cholesterol and ceramides, are known to be upregulated and secreted in an UPR-dependent way, affecting the recipient cells (17, 18, 19). In *Caenorhabditis elegans*, tyramine acts as a neurotransmitter downstream of neuronal IRE1α–XBP1 pathway to coordinate metabolism in intestine (20). The ability of the UPR in secretome alteration greatly expands its physiological function from single cell level to the organism level.

While most secretory proteins are transported through the ER-Golgi apparatus, some cytosolic proteins could be secreted *via* alternative ways, either through pores forming on plasma membrane, *via* plasma membrane budding, or by endocytic vesicle transport (21). Among these “unconventional” secretory routes, exosome secretion has received much attention. Exosomes are endosome-derived extracellular vesicles (EVs) with a diameter of about 30 to 150 nm. Different from microvesicles, another type of EVs that are generated by direct budding of the plasma membrane, exosomes originate from intraluminal vesicles (ILVs), which are small membrane vesicles formed by inward membrane budding in late endosomes. When the membrane of ILV-containing late endosomes, that is, multivesicular bodies (MVBs), fuse with plasma membrane, ILVs are released to extracellular spaces as exosomes (22). In addition to proteins, exosomes also comprise lipids, nucleic acids, metabolites, and small molecules, playing important roles in cargo delivery and signal transduction between cells (23).

As exosome secretion is tightly associated with the endomembrane system, it is an interesting question whether and how perturbation of the ER, a crucial component of this system, affects exosome secretion. The answer to this question, however, remains obscure, partly because many studies do not distinguish endosome-derived exosomes and other types of EVs. In addition, while some report showed that ER stress promotes exosome secretion in an IRE1α- and PERK-dependent way but with the mechanism unknown, other study suggested that it is lipotoxic stress caused by palmitate, rather than ER stress, that induces EVs release (24, 25). Palmitate treatment reduces ER membrane fluidity and activates the UPR in an ER stress–independent way (26, 27). In palmitate-treated hepatocytes, activation of IRE1α–XBP1 pathway promotes ceramide biosynthesis and ceramide-enriched EVs secretion, inducing inflammation in mice with steatohepatitis (28, 29).

Here, we report that ER stress promotes endosome-derived EV (*i.e.*, exosome) secretion in mouse melanoma cells. Both IRE1α and PERK are responsible for the upregulation of exosome release. Interestingly, this is achieved by reducing the acidity of lysosomes, which normally degrade MVBs. Our study suggests that the UPR signal stemming from the ER could modulate the degradation-secretion balance in the progression of vesicle transport.

## Results

### Both IRE1α and PERK contribute to EVs secretion under ER stress

**Figure 1:**
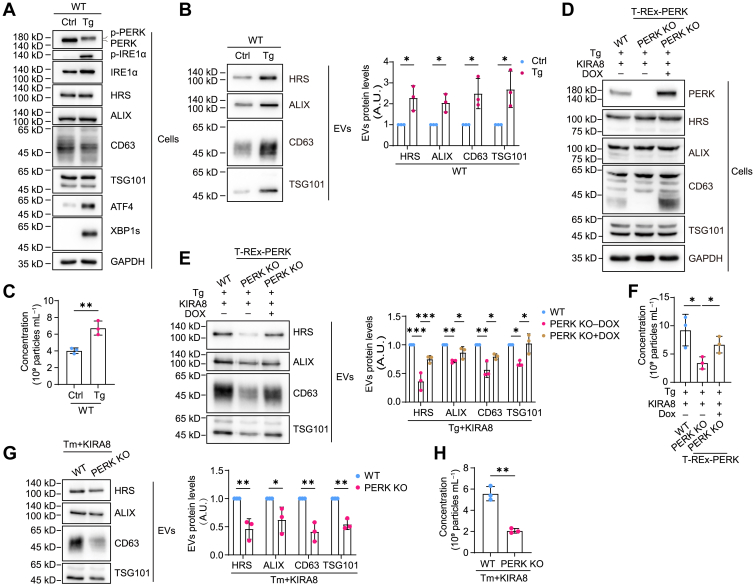
**PERK deficiency inhibits EVs secretion in ER-stressed cells pretreated with IRE1α inhibitor.***A*, Western blot analysis of UPR marker proteins and EVs marker proteins in WT B16.F10 cells treated with DMSO (Ctrl) or 10 nM Tg for 24 h. *B*, *left*, Western blot analysis of EVs purified from cell culture supernatants from equal numbers of WT B16.F10 cells treated as in (*A*). *Right*: quantification of band intensities of EVs marker proteins in EVs enriched from three independent experiments. *C*, quantification of EVs concentration by nanoparticle tracking analysis (NTA) (n = 3), normalized to cell number. EVs were obtained as in (*B*). *D*, Western blot analysis of UPR marker proteins and EVs marker proteins in WT, T-REx-PERK (endogenous PERK KO background), and Dox-pretreated T-REx-PERK (endogenous PERK KO background) B16.F10 cells treated with 10 nM Tg and 500 nM KIRA8 for 24 h. *E*, *left*: Western blot analysis of EVs purified from cell culture supernatants from equal numbers of WT, T-REx-PERK, and Dox-pretreated T-REx-PERK B16.F10 cells treated as in (*D*). *Right*: quantification of band intensities of EVs marker proteins in EVs enriched from three independent experiments. *F*, quantification of EVs concentration by NTA (n = 3), normalized to cell number. EVs were obtained as in (*E*). *G*, *left*: Western blot analysis of EVs purified from cell culture supernatants from equal numbers of WT and PERK KO B16.F10 cells treated with 0.5 μg/ml Tm and 500 nM KIRA8 for 24 h. *Right*: quantification of band intensities of EVs marker proteins in EVs enriched from three independent experiments. *H*, quantification of EVs concentration by NTA (n = 3), normalized to cell number. EVs were obtained as in (*G*). Statistical analysis was calculated by unpaired *t* test analysis, two tailed (*B*, *C*, *G*, and *H*) or one-way ANOVA (*E* and *F*). Data were shown as mean ± SD. ∗*p* < 0.05, ∗∗*p* < 0.01, and ∗∗∗*p* < 0.001. ER, endoplasmic reticulum; EV, extracellular vesicle; Tg, thapsigargin; Tm, tunicamycin; UPR, unfolded protein response.

We used thapsigargin (Tg), inhibitor of SERCA, to induce ER stress in mouse melanoma cell line B16.F10. Tg at 10 nM robustly activated both IRE1α and PERK pathway, evidenced by the phosphorylation of IRE1α and PERK as well as the expression of downstream transcription factors, XBP1s and ATF4, respectively (Fig. 1*A*). Meanwhile, Tg treatment elevated EVs generation (Fig. 1, *B* and *C*). IRE1α KO reduced EVs secretion level, whereas PERK deletion did not (Fig. S1, *A*–*F*). Proteomic analysis showed that isolated EVs were mainly derived from the endocytosis pathway, thus could be regarded as exosomes (Fig. S2). We found that XBP1 was upregulated upon PERK deletion, suggesting that IRE1α may compensate for PERK in EVs generation in the context of ER stress (Fig. S1*D*). Therefore, we pretreated cells with IRE1α inhibitor KIRA8, and compared EVs secretion level in WT or PERK KO cells. We found that PERK KO reduced the amount of EVs without altering the size distribution of EVs when IRE1α was inhibited, and re-expression of PERK restored EVs production, suggesting that both IRE1α and PERK pathways contribute to EVs secretion under ER stress, yet the role of PERK is masked by IRE1α (Figs. 1, *D*–*F* and S1*G*). Using another ER stress inducer, tunicamycin, which blocks protein N-glycosylation, we observed the same result that PERK deletion impaired EVs production in IRE1α-deficient cells (Fig. 1, *G* and *H*).

### PERK deletion promotes MVB degradation by lysosome in IRE1α-deficient cells that experience ER stress

**Figure 2:**
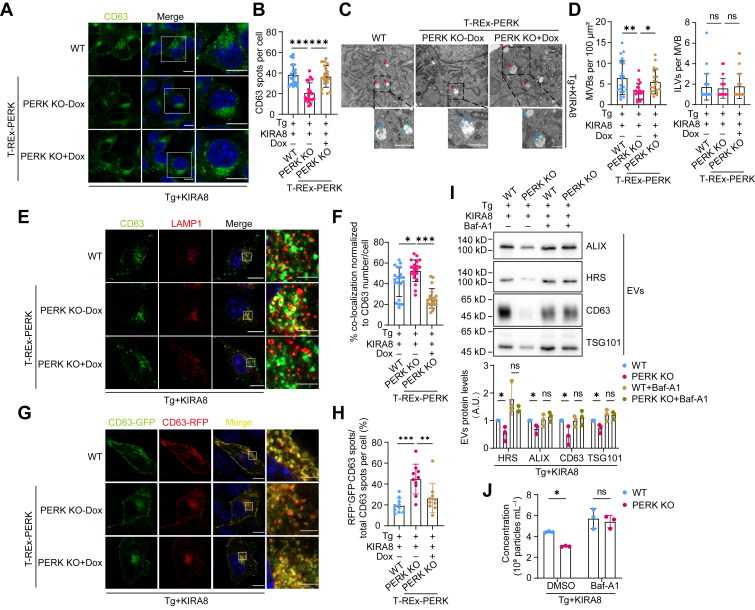
**PERK deletion promotes MVB degradation by lysosome in IRE1α-deficient cells that experience ER stress.***A*, confocal microscopy analysis of the MVB marker CD63 in WT, T-REx-PERK, and Dox-pretreated T-REx-PERK B16.F10 cells treated with 10 nM Tg and 500 nM KIRA8 for 6 h. The scale bar represents 10 μm. *B*, quantification of the number of CD63-positive spots per cell, n = 20. *C*, electron microscopy images of MVBs (*red arrows*) and ILVs (*blue arrows*) in WT, T-REx-PERK, and Dox-pretreated T-REx-PERK B16.F10 cells treated as in (*A*). The scale bar represents 500 nm. *D*, *left*: quantification of the number of MVBs per cell per 100 μm^2^ cytoplasm. Each dot represents the number of MVBs per section, n = 20. *Right*: quantification of the number of ILVs per MVB, n = 20. *E*, confocal microscopy analysis of CD63 and LAMP1 colocalization in WT, T-REx-PERK, and Dox-pretreated T-REx-PERK B16.F10 cells treated with 10 nM Tg and 500 nM KIRA8 for 6 h. The scale bar represents 10 μm (wide-view images) and 2 μm (enlarged images). *F*, quantification of the percentage of colocalized CD63 spots among the total CD63 spots per cell, n = 20. *G*, confocal microscopy analysis of RFP-GFP-CD63 endosomes in WT, T-REx-PERK, and Dox-pretreated T-REx-PERK B16.F10 cells treated with 10 nM Tg and 500 nM KIRA8 for 6 h. The scale bar represents 10 μm (wide-view images) and 2 μm (enlarged images). *H*, the percentage of RFP^+^GFP^−^ spots among the total RFP^+^ spots per cell. Each dot indicates the proportion of red-only CD63 spots per cell, n = 10. *I*, *up*: Western blot analysis of EVs derived from equal numbers WT and PERK KO B16.F10 cells treated with 10 nM Tg and 500 nM KIRA8 in the absence or presence of 40 nM bafilomycin A1 for 24 h. *Down*: quantitative analysis of band intensities of EVs marker proteins in EVs enriched from three independent experiments. *J*, quantification of EVs concentration by NTA (n = 3), normalized to cell number. EVs were obtained as in (*I*). Statistical analysis was calculated by one-way ANOVA (*B*, *D*, *F*, *H*, and *I*) or two-way ANOVA (*J*). Data were shown as mean ± SD. ns, not significant; ∗*p* < 0.05, ∗∗*p* < 0.01, and ∗∗∗*p* < 0.001. ER, endoplasmic reticulum; EV, extracellular vesicle; ILV, intraluminal vesicle; MVB, multivesicular body; NTA, nanoparticle tracking analysis; Tg, thapsigargin.

Since IRE1α-XBP1 signal is known to induce the biosynthesis of ceramide, which can drive the invert budding of endosome membrane to generate MVBs, we surmized that this is the reason why IRE1α contributes to EVs secretion (we will provide another mechanism later). Therefore, instead of delving into IRE1α-EVs secretion relationship, we first focused on the mechanism how PERK contributes to EVs secretion. Since PERK deletion reduced the cellular level of CD63 (Figs. 1*D* and S1*D*), we examined the role of CD63 in EVs production and found that EVs amount remained the same upon knocking down CD63, suggesting that the impairment of EVs secretion is not due to CD63 decrease (Fig. S3). Nevertheless, the decrease in CD63 as a biomarker of MVB suggested that PERK deletion may reduce the number of MVBs in cells that are under ER stress and have IRE1α pathway blocked. Both immunofluorescence imaging and electron microscope analysis supported this hypothesis. In addition, reintroducing PERK restored the number of MVBs (Fig. 2, *A*–*D*). The number of ILV per MVB was not changed upon PERK deletion or re-expression, indicating that PERK was dispensable for ILV formation (Fig. 2*D*). The decrease of MVBs upon PERK KO was less apparent in cells without IRE1α inhibition, implying that IRE1α may compensate for PERK in MVB formation or maintenance, in agreement with a previous study that XBP1s drives ceramide biosynthesis and MVB formation (29) (Fig. S4, *A* and *B*).

**Figure 3:**
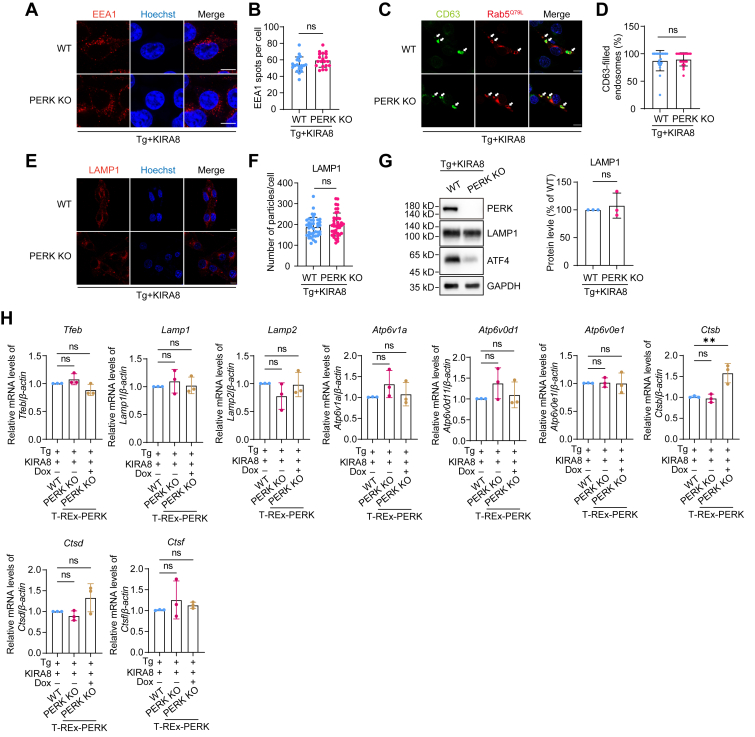
**PERK depletion does not affect the formation of early endosomes, the maturation of MVBs, the number of lysosomes, or lysosome biogenesis in IRE1α-deficient cells that experience ER stress.***A*, confocal microscopy analysis of the number of early endosomes marked by EEA1 in WT and PERK KO B16.F10 cells treated with 10 nM Tg and 500 nM KIRA8 for 6 h. The scale bar represents 10 μm. *B*, quantification of the number of EEA1-positive fluorescent spots. Each dot represents the number of EEA1-positive fluorescent spots per cell, n = 20. *C*, confocal microscopy analysis of CD63 sorting into Rab5^Q79L^-mCherry endosomes. WT and PERK KO B16.F10 cells were transiently transfected with Rab5^Q79L^-mCherry plasmid for 24 h, followed by treatment with 10 nM Tg and 500 nM KIRA8 for 6 h. *White arrows* indicate endosomes containing CD63 signal. The scale bar represents 10 μm. *D*, quantification of the percentage of CD63-filled endosomes per cell, n = 20. *E*, confocal microscopy analysis of LAMP1 in WT and PERK KO B16.F10 cells treated with 10 nM Tg and 500 nM KIRA8 for 6 h. The scale bar represents 10 μm. *F*, quantification of the number of LAMP1-positive spots per cell, n = 20. *G*, *left*: Western blot analysis of LAMP1 in WT and PERK KO B16.F10 cells treated with 10 nM Tg and 500 nM KIRA8 for 6 h. *Right*, quantification of the LAMP1 protein levels in cells, with data representing results from three independent experiments. *H*, qPCR analysis of lysosome biogenesis-related genes in WT, T-REx-PERK, and Dox-pretreated T-REx-PERK B16.F10 cells treated with 10 nM Tg and 500 nM KIRA8 for 6 h (data from three independent experiments). Statistical analysis was calculated by unpaired *t* test analysis, two tailed (*B*, *D*, *F*, and *G*) or one-way ANOVA (*H*). Data were shown as mean ± SD. ns, not significant. ER, endoplasmic reticulum; MVB, multivesicular body; qPCR, quantitative PCR; Tg, thapsigargin.

The decrease in MVBs number could be due to impairment of MVB formation, upregulation in MVBs degradation, or elevated fusion of MVBs with plasma membrane. Given that the number of ILVs per MVB was not changed, the elevated fusion of MVBs with plasma membrane would result in more EVs secretion, which contradicted with our observation and thus could be ruled out. As for MVB formation, we examined early endosomes, the origin of MVBs, and found that PERK KO did not reduce the number of early endosomes (Fig. 3, *A* and *B*). Rab5^Q79L^ is a constitutively active mutant that can facilitate the observation of endosomes (30, 31). The number of CD63-filled endosomes, indicating the formation of MVBs, was not affected by PERK KO (Fig. 3, *C* and *D*). These results, together with the fact that the number of ILVs per MVB was not changed upon PERK deletion (Fig. 2, *C* and *D*), suggest that the decrease of EVs was not due to impairment of MVB formation.

MVBs could either fuse with plasma membrane, releasing ILVs as EVs, or undergo degradation after fusion with lysosome. Notably, PERK deletion promoted the colocalization of CD63 and LAMP1, a biomarker of lysosome. Re-expression of PERK jeopardized such colocalization (Fig. 2, *E* and *F*). Furthermore, using CD63 tandemly fused with RFP and GFP as a reporter of lysosome localization, we observed higher ratio of RFP to GFP in cells with PERK KO, demonstrating that PERK deletion promoted MVB and lysosome fusion in cells that are under ER stress and have the IRE1α pathway inhibited (Fig. 2, *G* and *H*). Next, we blocked lysosomal function using a vacuolar H^+^-ATPase inhibitor, bafilomycin A1. This greatly restored EVs secretion (Fig. 2, *I* and *J*). Therefore, in ER stress–challenged and IRE1α-deficient cells, PERK deletion promotes MVB-lysosome fusion, resulting in MVBs degradation and the consequent impairment in EVs secretion. Again, PERK KO did not increase MVB-lysosome fusion if IRE1α activity was intact, indicating that IRE1α may negatively regulate MVB-lysosome fusion (Videos S1-S2 and Fig. S4*C*).

### Lysosome activity is enhanced upon PERK deletion in IRE1α-deficient cells that experience ER stress

**Figure 4:**
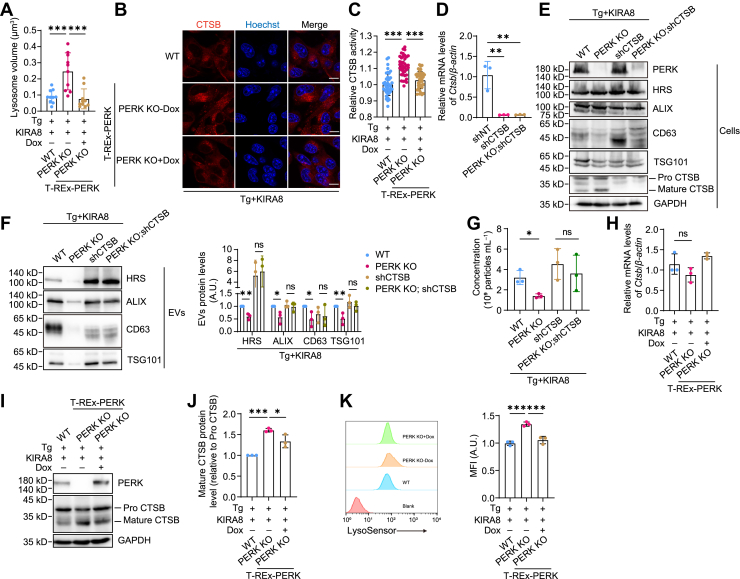
**Lysosome activity is enhanced upon PERK deletion in IRE1α-deficient cells that experience ER stress.***A*, quantification of lysosome volume in WT, T-REx-PERK, and Dox-pretreated T-REx-PERK B16.F10 cells treated with 10 nM Tg and 500 nM KIRA8 for 6 h based on the 3D result in Videos S3–S5. The scale bar represents 10 μm. *B*, confocal imaging of WT, T-REx-PERK, and Dox-pretreated T-REx-PERK B16.F10 cells stained with Magic *Red* to assess CTSB activity within lysosomes after treatment with 10 nM Tg and 500 nM KIRA8 for 6 h. The scale bar represents 10 μm. *C*, quantification of fluorescence intensity in Magic *Red*–stained cells using ImageJ. Each dot represents the mean fluorescence intensity per cell, n = 40. *D*, qPCR analysis of CTSB knockdown efficiency at the mRNA level in shNT and shCTSB B16.F10 cells. *E*, Western blot analysis of EVs marker proteins in WT, PERK KO, shCTSB, and PERK KO-shCTSB B16.F10 cells following treatment with 10 nM Tg and 500 nM KIRA8 for 24 h. *F*, *left*: Western blot analysis of EVs purified from cell culture supernatants from equal numbers of WT, PERK KO, shCTSB, and PERK KO-shCTSB B16.F10 cells following treatment with 10 nM Tg and 500 nM KIRA8 for 24 h. *Right*: quantification of EVs marker protein levels in the EVs obtained from cell culture supernatants of WT, PERK KO, shCTSB, and PERK KO-shCTSB B16.F10 cells treated with 10 nM Tg and 500 nM KIRA8 for 24 h. The figure shows the results from three independent experiments. *G*, quantification of EVs concentration by NTA (n = 3), normalized to cell number. EVs were obtained as in (*F*). *H*, qPCR analysis of *ctsb* expression levels in WT, T-REx-PERK, and Dox-pretreated T-REx-PERK B16.F10 cells treated with 10 nM Tg and 500 nM KIRA8 for 6 h. *I*, Western blot analysis of CTSB expression in WT, T-REx-PERK, and Dox-pretreated T-REx-PERK B16.F10 cells treated with 10 nM Tg and 500 nM KIRA8 for 6 h. *J*, quantitative analysis of the relative abundance of the mature form of CTSB in cells, with data representing results from three independent experiments. *K*, flow cytometric analysis of LysoSensor mean fluorescence intensity (MFI) in WT, T-REx-PERK, and Dox-pretreated T-REx-PERK B16.F10 cells treated with 10 nM Tg and 500 nM KIRA8 for 6 h to evaluate lysosomal acidity. The *left panel* shows a representative result, and the *right panel* presents statistical data from three independent experiments. Statistical analysis was calculated by one-way ANOVA. Data were shown as mean ± SD. ns, not significant; ∗*p* < 0.05, ∗∗*p* < 0.01, and ∗∗∗*p* < 0.001. CTSB, cathepsin B; ER, endoplasmic reticulum; EV, extracellular vesicle; MVB, multivesicular body; NTA, nanoparticle tracking analysis; qPCR, quantitative PCR; Tg, thapsigargin.

We have shown that PERK plays important roles in EVs secretion by regulating the transport route of MVBs. Next, we examined whether the number and the activity of lysosomes are enhanced in PERK KO cells. In the presence of IRE1α inhibitor, PERK deletion did not reduce the number of lysosomes in cells with ER stress (Fig. 3, *E* and *F*). Protein level of LAMP1 was also unaffected (Fig. 3*G*). In addition, PERK KO had no effect on the expression level of lysosome biogenesis-related genes, including those transcribes transcription factor (*Tfeb*), lysosome markers (*Lamp1* and *Lamp2*), proton pump (*Atp6v1a*, *Atp6v0d1,* and *Atp6v0e1*), and lysosomal proteases (*Ctsb*, *Ctsd,* and *Ctsf*) (Fig. 3*H*). On the other hand, the size of lysosomes was enlarged in PERK KO cells and could be brought back by PERK overexpression (Fig. 4*A* and Videos S3–S5). Lysosomal cathepsin B (CTSB) activity, measured by Magic Red staining, was greatly improved upon PERK KO, and was suppressed by PERK re-expression (Fig. 4, *B* and *C*). Knocking down CTSB, one of the major lysosomal cathepsins, enhanced EVs secretion and undermined its dependence on PERK (Fig. 4, *D*–*G*). We then analyzed the expression level of CTSB, and found that CTSB was not upregulated at transcription level upon PERK deletion; rather, the mature form of CTSB became more pronounced when PERK is deleted, which was reduced again upon PERK overexpression (Fig. 4, *H*–*J*). The processing of pro-CTSB occurs in the lysosome in an acidic condition–dependent way (32). In line with this, lysosomes in PERK KO cells exhibited higher acidity than that in WT cells, and PERK overexpression reduced the acidity level (Fig. 4*K*). Taken together, PERK deletion increased catabolic activity and the acidity level in lysosomes, which are then more avid to degrade MVBs. It should be noted that the role of PERK could be compensated by IRE1α, as the lysosome acidity was not increased by PERK KO if IRE1α remained active (Fig. S4*D*).

### ATF4 promotes EVs secretion through downregulating lysosome activity

**Figure 5:**
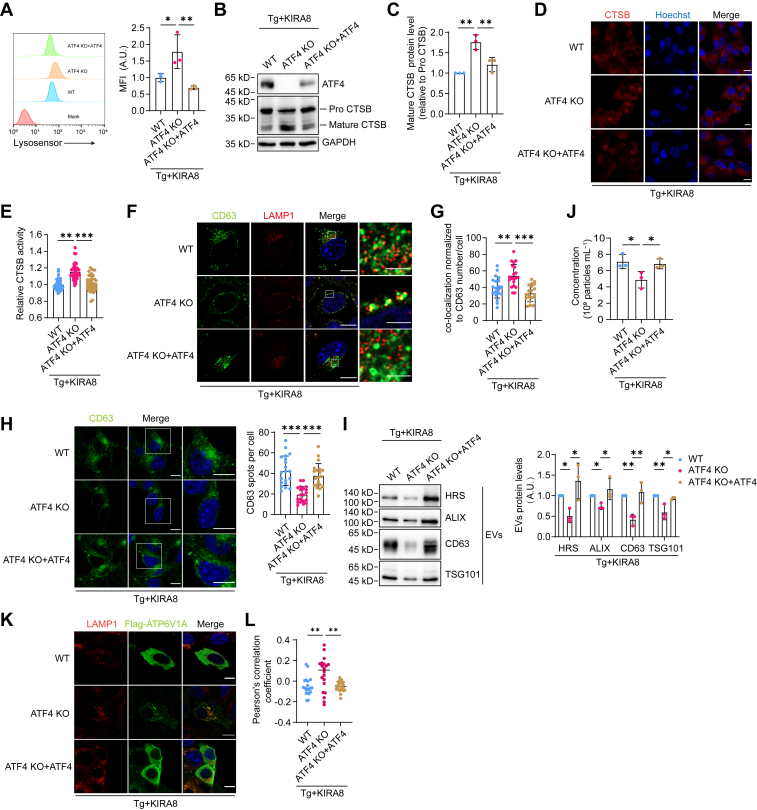
**ATF4 promotes EVs secretion through downregulating lysosome activity.***A*, flow cytometric analysis of LysoSensor mean fluorescence intensity (MFI) in WT, ATF4 KO, and ATF4-rescued ATF4 KO B16.F10 cells treated with 10 nM Tg and 500 nM KIRA8 for 6 h to evaluate lysosomal acidity. The *left panel* shows a representative result, and the *right panel* presents statistical data from three independent experiments. *B*, Western blot analysis of CTSB expression in WT, ATF4 KO, and ATF4-rescued ATF4 KO B16.F10 cells following treatment with 10 nM Tg and 500 nM KIRA8 for 6 h. *C*, quantitative analysis of the relative abundance of the mature form of CTSB in cells, with data representing results from three independent experiments. *D*, confocal imaging of WT, ATF4 KO, and ATF4-rescued ATF4 KO B16.F10 cells stained with Magic Red to assess CTSB activity within lysosomes after treatment with 10 nM Tg and 500 nM KIRA8 for 6 h. The scale bar represents 10 μm. *E*, quantification of fluorescence intensity in Magic Red–stained cells using ImageJ. Each dot represents the mean fluorescence intensity per cell, n = 40. *F*, confocal microscopy analysis of CD63 and LAMP1 colocalization in WT, ATF4 KO, and ATF4-rescued ATF4 KO B16.F10 cells treated with 10 nM Tg and 500 nM KIRA8 for 6 h. The scale bar represents 10 μm (wide-view images) and 2 μm (enlarged images). *G*, quantification of the percentage of LAMP1 colocalized CD63 spots among the total CD63 spots per cell, n = 20. *H*, *left*: confocal microscopy analysis of the MVB marker CD63 in WT, ATF4 KO, and ATF4-rescued ATF4 KO B16.F10 cells treated with 10 nM Tg and 500 nM KIRA8 for 6 h. The scale bar represents 10 μm. *Right*: Quantification of the number of CD63-positive spots per cell, n = 20. *I*, *left*: Western blot analysis of EVs purified from cell culture supernatants from equal numbers of WT, ATF4 KO, and ATF4-rescued ATF4 KO B16.F10 cells following treatment with 10 nM Tg and 500 nM KIRA8 for 24 h. *Right*: quantification of EVs marker protein levels in the EVs obtained from cell culture supernatants of WT, ATF4 KO, and ATF4-rescued ATF4 KO B16.F10 cells treated with 10 nM Tg and 500 nM KIRA8 for 24 h. The figure shows the results from three independent experiments. *J*, quantification of EVs concentration by NTA (n = 3), normalized to cell number. EVs were obtained as in (*I*). *K*, confocal microscopy analysis of the colocalization of ATP6V1A and LAMP1. WT, ATF4 KO, and ATF4-rescued ATF4 KO B16.F10 cells were transiently transfected with Flag-ATP6V1A for 24 h and subsequently treated with 10 nM Tg and 500 nM KIRA8 for 6 h. Flag-ATP6V1A was stained by anti-Flag antibody (*green*). The scale bar represents 10 μm. *L*, Pearson’s correlation coefficients for Flag-ATP6V1A and LAMP1 colocalization, n = 20. Statistical analysis was calculated by one-way ANOVA. Data were shown as mean ± SD. ∗*p* < 0.05, ∗∗*p* < 0.01, and ∗∗∗*p* < 0.001. CTSB, cathepsin B; EV, extracellular vesicle; MVB, multivesicular body; NTA, nanoparticle tracking analysis; Tg, thapsigargin.

It is intriguing to understand how PERK regulates lysosome activity. ATF4 is a transcription factor that can be translationally upregulated by PERK and has been known to regulate autophagy (33, 34). We found that PERK deletion impaired ER stress–induced ATF4 upregulation (Fig. S5*A*). We then generated ATF4 KO cells. Interestingly, ATF4 deletion increased the level of lysosome acidity, CTSB maturation and activity, and promoted MVBs-lysosome fusion, leading to the decrease in MVBs number and EVs secretion. All of the above effects could be restored by ATF4 overexpression (Fig. 5, *A*–*J*). Furthermore, replenishment of ATF4 in PERK KO cells improved EVs secretion (Fig. S5, *B* and *C*). Thus, ATF4 is the key factor participating in PERK-regulated lysosome activation and EVs secretion.

We further explored how ATF4 negatively regulates lysosome activities. The mRNA levels for genes involved in lysosome biogenesis were not elevated upon ATF4 deletion, denying transcriptional regulation by ATF4 (Fig. S5*D*). Vacuolar-type H^+^ ATPase (V-ATPase) pumps protons into the lysosome and is essential for lysosome acidification. Recruitment of a peripheral V_1_ domain of V-ATPase to the lysosome membrane where V_0_ domain resides enables assembly of active V-ATPase (35, 36). Notably, the colocalization of V1 and LAMP1 was enhanced by ATF4 deletion and was brought down again when ATF4 was re-expressed (Fig. 5, *K* and *L*). We thus concluded that ATF4 downregulates lysosome acidification by suppressing the assembly of V-ATPase.

### Activation of PERK reduced lysosome activity and augments EV secretion

**Figure 6:**
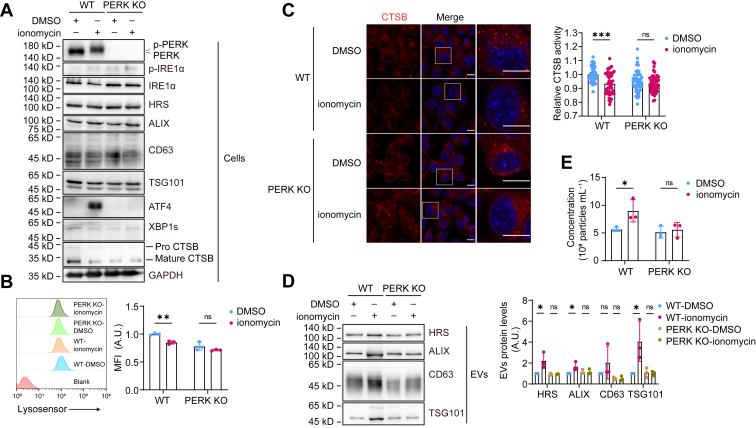
**Activation of PERK reduced lysosome activity and augments EVs secretion.***A*, Western blot analysis of cell lysates from WT and PERK KO B16.F10 cells treated with DMSO or 2 μM ionomycin for 3 h. *B*, flow cytometric analysis of LysoSensor mean fluorescence intensity (MFI) in WT and PERK KO B16.F10 cells treated with DMSO or 2 μM ionomycin for 3 h to evaluate lysosomal acidity. The *left panel* shows a representative result, and the *right panel* presents statistical data from three independent experiments. *C*, *left*: confocal imaging of WT and PERK KO B16.F10 cells stained with Magic Red to assess CTSB activity within lysosomes after treatment with DMSO or 2 μM ionomycin for 3 h. The scale bar represents 10 μm. *Right*: quantification of fluorescence intensity in Magic Red–stained cells using ImageJ. Each dot represents the mean fluorescence intensity per cell, n = 40. *D*, *left*: Western blot analysis of EVs enriched from equal numbers of WT and PERK KO B16.F10 cells treated with DMSO or 2 μM ionomycin for 3 h. *Right*: quantitative analysis of marker protein levels in EVs enriched from WT and PERK KO B16.F10 cells treated with DMSO or 2 μM ionomycin for 3 h. The figure shows results from three independent experiments. *E*, quantification of EVs concentration by NTA (n = 3), normalized to cell number. EVs were obtained as in (*D*). Statistical analysis was calculated by one-way ANOVA (*D*) or two-way ANOVA (*B*, *C*, and *E*). Data were shown as mean ± SD. ns, not significant; ∗*p* < 0.05, ∗∗*p* < 0.01, and ∗∗∗*p* < 0.001. CTSB, cathepsin B; EV, extracellular vesicle; MVB, multivesicular body; NTA, nanoparticle tracking analysis.

The above results showed that under ER stress, PERK–ATF4 pathway contributes to EVs production *via* regulating the MVBs transportation, suppressing its lysosome fusion and degradation while promoting the secretion. However, such effect could be masked by IRE1α, the activity of which was improved by PERK deletion. We wondered whether PERK activation could promote EVs production independent of IRE1α. Cytosolic Ca^2+^ directly binds to and activates PERK without activating IRE1α (37). We postulated that elevation of cytosolic Ca^2+^ concentration would be a physiological condition that induces EVs secretion through specifically activating PERK. We thus treated cells with ionomycin to elevate Ca^2+^ concentration in the cytosol. Ionomycin treatment for 3 h specifically activated PERK but not IRE1α. Notable, ionomycin reduced lysosome acidity and CTSB activity and promoted EVs production in a PERK-dependent way (Fig. 6, *A*–*E*). These results suggest that calcium-mediated PERK activation can decrease lysosome activity and promote MVB secretion independent of ER stress and IRE1α.

### IRE1α negatively regulates lysosome activity and MVB degradation

**Figure 7:**
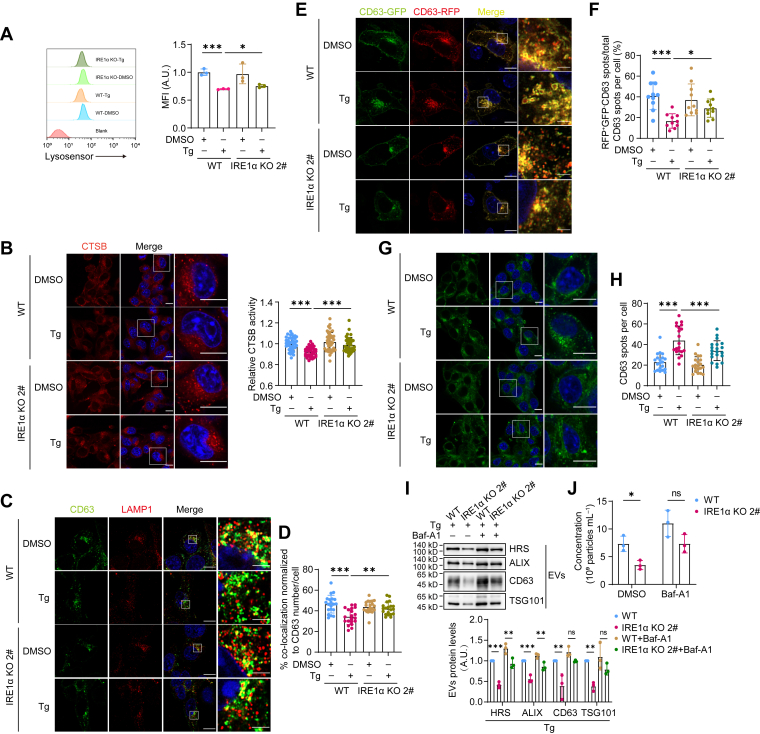
**IRE1α negatively regulates lysosome activity and MVB degradation.***A*, flow cytometric analysis of LysoSensor mean fluorescence intensity (MFI) in WT and IRE1α KO B16.F10 cells treated with DMSO or 10 nM Tg for 6 h to evaluate lysosomal acidity. The *left panel* shows a representative result, and the *right panel* presents statistical data from three independent experiments. *B*, *left*: confocal imaging of WT and IRE1α KO B16.F10 cells stained with Magic Red to assess CTSB activity within lysosomes after treatment with DMSO or 10 nM Tg for 6 h. The scale bar represents 10 μm. *Right*: quantification of fluorescence intensity in Magic Red–stained cells using ImageJ. Each dot represents the mean fluorescence intensity per cell, n = 40. *C*, confocal microscopy analysis of CD63 and LAMP1 colocalization in WT and IRE1α KO B16.F10 cells treated with DMSO or 10 nM Tg for 6 h. The scale bar represents 10 μm (wide-view images) and 2 μm (enlarged images). *D*, quantification of the percentage of colocalized CD63 spots among the total CD63 spots per cell, n = 20. *E*, confocal microscopy analysis of RFP-GFP-CD63 endosomes in WT and IRE1α KO B16.F10 cells treated with DMSO or 10 nM Tg for 6 h. The scale bar represents 10 μm (wide-view images) and 2 μm (enlarged images). *F*, the percentage of RFP^+^GFP^−^ spots among the total RFP^+^ spots per cell. Each dot indicates the proportion of red-only CD63 spots per cell, n = 10. *G*, confocal microscopy analysis of the MVB marker CD63 in WT and IRE1α KO B16.F10 cells treated with DMSO or 10 nM Tg for 6 h. The scale bar represents 10 μm. *H*, quantification of the number of CD63-positive spots per cell, n = 20. *I*, *up*: Western blot analysis of EVs enriched from equal numbers of WT and IRE1α KO B16.F10 cells treated with 10 nM Tg in the absence or presence of 40 nM bafilomycin A1 for 24 h. *Down*: quantitative analysis of marker protein levels in EVs enriched from WT and IRE1α KO B16.F10 cells treated with 10 nM Tg in the absence or presence of 40 nM bafilomycin A1 for 24 h. The figure shows results from three independent experiments. *J*, quantification of EVs concentration by NTA (n = 3), normalized to cell number. EVs were obtained as in (*I*). Statistical analysis was calculated by one-way ANOVA (*A*, *B*, *D*, *F*, *H*, and *I*) or two-way ANOVA (*J*). Data were shown as mean ± SD. ns, not significant; ∗*p* < 0.05, ∗∗*p* < 0.01, and ∗∗∗*p* < 0.001. CTSB, cathepsin B; EV, extracellular vesicle; MVB, multivesicular body; NTA, nanoparticle tracking analysis; Tg, thapsigargin.

**Figure 8:**
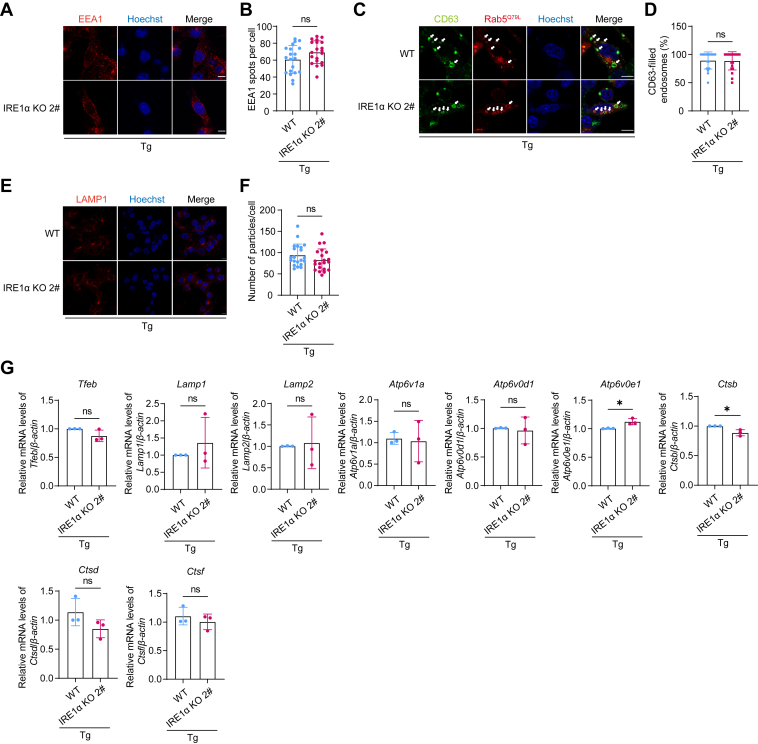
**IRE1α depletion does not affect the formation of early endosomes, the maturation of MVBs, the number of lysosomes, or lysosome biogenesis.***A*, confocal microscopy analysis of the number of early endosomes marked by EEA1 in WT and IRE1α KO B16.F10 cells treated with 10 nM Tg for 6 h. The scale bar represents 10 μm. *B*, quantification of the number of EEA1-positive fluorescent spots. Each dot represents the number of EEA1-positive fluorescent spots per cell, n = 20. *C*, confocal microscopy analysis of CD63 sorting into Rab5^Q79L^-mCherry endosomes. WT and IRE1α KO B16.F10 cells were transiently transfected with Rab5^Q79L^-mCherry plasmid for 24 h, followed by treatment with 10 nM Tg for 6 h. *White arrows* indicate endosomes containing CD63 signal. The scale bar represents 10 μm. *D*, quantification of the percentage of CD63-filled endosomes per cell, n = 20. *E*, confocal microscopy analysis of LAMP1 in WT and IRE1α KO B16.F10 cells treated with 10 nM Tg for 6 h. The scale bar represents 10 μm. *F*, quantification of the number of LAMP1-positive spots per cell, n = 20. *G*, qPCR analysis of lysosome biogenesis-related genes in WT and IRE1α KO cells treated with 10 nM Tg for 6 h (data from three independent experiments). Statistical analysis was calculated by unpaired *t* test analysis, two tailed. Data were shown as mean ± SD. ns, not significant; ∗*p* < 0.05. MVB, multivesicular body; qPCR, quantitative PCR; Tg, thapsigargin.

If IRE1α promotes EVs secretion only through upregulating ceramide biosynthesis and MVB formation, the compensatory activation of IRE1α in PERK KO cells should not mask the effect of PERK deletion on lysosome acidification and MVB-lysosome fusion, which contradicted our observations. Therefore, we investigated whether IRE1α also repressed lysosome activity and prevented MVB degradation. Compared to WT cells, IRE1α KO cells exhibited elevated lysosomal acidity and higher CTSB activity (Fig. 7, *A* and *B*). Similar to that in PERK KO cells, the fusion of MVBs and lysosomes was upregulated in IRE1α KO cells, with CD63-RFP-GFP reporter more likely to be transported to lysosomes (Fig. 7, *C*–*F*). As a result, less MVBs were observed in IRE1α KO cells compared to WT (Fig. 7, *G* and *H*). IRE1α deletion did not affect early endosome formation, MVB maturation, lysosome number, or lysosome biogenesis (Fig. 8). Furthermore, inhibition of lysosome with bafilomycin A1 revived EVs secretion in IRE1α KO cells, supporting the idea that IRE1α deletion impaired EVs secretion through activation of lysosomal degradation of MVBs (Fig. 7, *I* and *J*). Thus, both IRE1α and PERK can reduce the lysosome acidity and activity, rendering more MVB secretion while inhibiting its lysosomal degradation.

## Discussion

### The UPR can regulate exosome secretion through different mechanisms

Cell-to-cell communication *via* EVs are prevalent and important under numerous (patho)physiological conditions. The biogenesis of exosome, one type of EVs, includes steps of ILV formation (endosomal membrane invagination and scission) and ILV release (MVB-plasma membrane fusion). Previous studies found that palmitate treatment or FFC (fat, fructose, glucose) diet induced EVs secretion in hepatocyte and in mice, respectively, through IRE1α–XBP1 pathway that upregulates ceramide production (28, 29). Moreover, palmitate treatment augmented ceramide content in EVs secreted from hepatocyte (28). With a cone-shaped structure, ceramide is known to generate negative lipid membrane curvature and has been reported to trigger endosomal membrane invagination and ILV formation (38). These studies indicate that IRE1α can increase exosome production by promoting budding of ceramide-rich subdomains on the endosomal membrane for ILV formation. Here, we proved that under Tg-induced ER stress, IRE1α advances exosome secretion *via* reducing lysosomal degradation of MVBs. Thus, IRE1α may induce exosome production *via* multiple mechanisms. Similarly, PERK also induces exosome secretion through repressing MVB degradation in Tg- or ionomycin-treated cells, highlighting the role of the UPR in the regulation of endosome trafficking and exocytosis.

### How does the UPR regulate lysosome acidity?

We found that Tg-induced ER stress reduced lysosome acidity in an IRE1α- and PERK-dependent manner. In addition, ionomycin suppressed lysosomal acidification PERK dependently. Both Tg and ionomycin can increase Ca^2+^ concentration in cytosol ([Ca^2+^]_cyto_), raising the possibility of [Ca^2+^]_cyto_-regulated lysosome acidification. This hypothesis could be tested by using Ca^2+^ chelators. A previous study showed that Ca^2+^ inhibited acidification of yeast vacuole at micromolar levels (39). Whether this applies in mammalian cells and what the mechanism is remain unknown and require further study. Recently, we discovered that Ca^2+^ can interact with and activate PERK in an ER stress–independent way (37). Here, we showed that ionomycin specifically activated PERK, and PERK deletion restored lysosomal acidification. Taken together, it is reasonable to postulate that [Ca^2+^]_cyto_ elevation can compromise lysosomal acidification *via* activating PERK. Since [Ca^2+^]_cyto_ elevation could happen under many conditions, for example, virus infection and insulin signaling (40, 41), the regulation of lysosome pH by PERK may widely exist.

Specifically, the regulation of PERK on lysosome activity and acidification depends on its downstream protein ATF4, whose deletion triggered the recruitment of V-ATPase V1A subunit to lysosome membrane. This resembles the observation in a previous study that V1 domain was recruited to lysosome to assemble active proton pumps when mTORC1 activity declined (42). ATF4 is well known for its important role in amino acid import and metabolism (43). Thus, ATF4 KO may result in amino acid starvation, inactivating mTORC1. As for IRE1α, we also saw elevated lysosome acidity and activity upon its deletion. So far, the mechanism remains largely unknown, except that IRE1α deletion did not upregulate lysosome biogenesis transcriptionally, which was also the case in PERK or ATF4 deletion. How the UPR regulates lysosome acidity and activity is an interesting question to be solved.

### The relationship between lysosome acidification and lysosome-MVB fusion

We found that deletion of either PERK, ATF4, or IRE1α increased lysosome acidity and CTSB activity, meanwhile promoting lysosome-MVB fusion and CD63-GFP signal quenching. Lysosome acidification is critical for its catabolic activity and is thought to be linked with its fusion with autophagosome (42, 44). However, a study using *Drosophila* showed that genetic depletion of V-ATPase subunits disrupted lysosomal acidity but had no effect on lysosome-autophagosome fusion (45). Although not examined for lysosome-MVB fusion, this study suggests that the role of lysosome acidification in lysosome-MVB fusion is not self-evident and needs to be examined. Three possibilities exist. First, lysosomal acidification directly promotes lysosome-MVB fusion. Second, lysosome-MVB fusion is reversible, and lysosomes with higher acidity are more prone to degrade MVBs, resulting in equilibrium shift that promotes the fusion. In this model, ILVs may still have the chance to escape from lysosomes after the latter fused with MVBs. This might be achieved by lysosomal exocytosis (46). Third, the increase of lysosome-MVB fusion is not due to elevated lysosome acidification. In this case, augmentations in both lysosome-MVB fusion and lysosomal acidification contribute to ILV degradation independently. In either way, the elevation in lysosome acidity and activity can promote MVB degradation and jeopardize exosome release.

### The significance of UPR-induced exosome secretion

ER stress is characterized by protein misfolding and unfolded protein accumulation in the ER lumen, which is usually accompanied by blockage in conventional protein secretion and elevated protein degradation (47, 48). EVs play important roles in unconventional protein secretion, which is largely triggered by cellular stresses (21). Under cellular stress, UPR-induced EVs secretion could likely compensate for the reduction in conventional protein secretion, meanwhile transmitting stress signals to other cells. Besides, the UPR also regulates lipid metabolism (13). Thus, its involvement in EVs formation facilitates adjustment of lipid composition in EVs, which have been proved for ceramide and cholesterol (17, 28). We found that both IRE1α and PERK induce EVs secretion by re-routing MVBs destination from lysosomal degradation to plasma fusion through decreasing lysosomal activities. This seems unpredicted at the first glance, considering that protein degradation capacity should be highly demanded during ER stress. However, ER stress can trigger ER-associated degradation that delivers misfolded proteins to proteasome for degradation, compensating for the decrease in lysosomal degradation (47). Moreover, reducing lysosomal activity can augment not only exosome secretion but also, in principle, secretory autophagy. If these are true, materials in autophagosomes, which may come from the ER, can be expelled from the cell without degradation, serving in unconventional secretion as well as stress signal broadcast intercellularly.

PERK pathway is dispensable in ER stress-induced exosome secretion, since the compensatory activation of IRE1α is sufficient to induce exosome release. RPAP2, a phosphatase downstream of PERK, has been reported to dephosphorylate IRE1α (49). This may explain why IRE1α-XBP1 signal was upregulated in PERK KO cells. Though dispensable, the role of PERK in exosome release should not be neglected. PERK can be activated independent of ER stress, in which scenario IRE1α remains inactive (37). Ca^2+^ in the cytosol activates PERK, suggesting that PEKR is involved in Ca^2+^-regulated exosome secretion (37, 50). In addition, IRE1α may undergo inactivation through different ways, for example, S-nitrosylation and degradation *via* ER-associated degradation (51, 52). Impairment of IRE1α-XBP1 has been reported in certain type of cancer cells and is thought to contribute to tumor growth (53). Under such circumstances, the importance of PERK will be highlighted. Furthermore, ATF4 contributes to exosome release. ATF4 can be activated through multiple ways including PERK, GCN2, PKR, and HRI, which response to a variety of cellular stresses, constituting the integrated stress response (54). This broadens the way how exosome secretion is regulated under cellular stress. Finally, while both PERK and IRE1α induce exosome secretion through similar mechanism, they may probably regulate exosome cargo selection and modify the exosome lipid composition differently. Systematic comparison of exosome contents regulated by IRE1α and PERK is important for comprehensive understanding of the biological functions of the UPR-mediated exosome generation.

## Experimental procedures

### Cell culture

B16.F10 cells were cultured in Dulbecco's modified Eagle's medium (DMEM) containing 10% fetal bovine serum (PAN-Biotech, Cat#ST30-3320) and 100 μg/ml penicillin-streptomycin at 37 °C in a 5% CO_2_ humidified incubator.

### EVs isolation

Fetal bovine serum was centrifuged at 120,000*g* overnight for over 18 h to remove EVs. Then, it was added to DMEM to achieve a final concentration of 10% (v/v). B16.F10 cells were cultured in DMEM until approximate 90% confluence in 15 cm cell culture dishes. Then, the conditional culture medium was collected. Subsequently, a differential centrifugation method was used to separate EVs from the culture medium. All centrifugation steps were performed at 4 °C. First, the culture medium was centrifuged at 300*g* for 10 min to remove cells. Then, the cell-free supernatant was centrifuged at 2000*g* for 20 min to remove cell debris and apoptotic bodies. Following that, the supernatant was centrifuged at 10,000*g* for 40 min to remove large EVs. To eliminate any remaining large EVs, the media supernatant was passed through a 0.22 μm pore polyethersulfone filter (Millipore, Cat#SLGP033RB). Next, the supernatant was subjected to ultracentrifugation at 120,000*g* for 2 h in an SW28 swinging bucket rotor (Beckman Coulter) to pellet the EVs. The crude EVs pellet was resuspended in a large volume of ice-cold PBS and subjected to an additional 120,000*g* centrifugation for 2 h to wash the EVs. Finally, the pellet was resuspended in ice-cold PBS.

### Nanoparticle tracking analysis

The NS300 instrument (Malvern) equipped with a 488 nm laser and a high-sensitivity sCMOS camera was employed for the precise measuring and determining the size of EVs. Particles were automatically traced and their sizes were measured based on Brownian motion and diffusion coefficients. EVs were resuspended and appropriately diluted in PBS, followed by thorough vortex mixing. Subsequently, the samples were loaded into the sample chamber at room temperature. For each sample, three 60 s videos were recorded. These videos were later analyzed using NTA3.3 software (https://www.malvernpanalytical.com.cn/support/product-support/software/nanosight-nta-software-update-v3-3).

### Electron microscope

For EVs negative staining, the copper grids were first subjected to glow discharge treatment. Subsequently, 5 μl of freshly isolated EVs samples were dropped onto the copper grids, allowed to adsorb for 1 min, and then blotted with filter paper to remove excess sample. The grids were rinsed twice with uranyl acetate and excess uranyl acetate was removed by blotting with filter paper. Then, 8 μl of uranyl acetate stain was added and left for 1 min before excess stain was removed with filter paper. After air-drying, the copper grids were analyzed by conventional electron microscopy.

For MVB analysis, B16.F10 cells were fixed with 2.5% (v/v) glutaraldehyde in PBS overnight at 4 °C. The samples were washed three times in PBS for 10 min each and then fixed in 1% osmium tetroxide for 90 min at room temperature. Subsequently, the samples were dehydrated through a graded ethanol series (30%, 50%, 70%, 80%, 90%, and 100%) and then in 100% acetone for 10 min. The samples were subsequently immersed in a series of acetone and resin mixtures at varying ratios (3:1, 1:1, and 1:3) before transitioning to pure resin. Following this, the cells were embedded in pure resin and polymerized for 12 h at 45 °C, followed by an additional 48 h at 60 °C. Ultrathin sections were then prepared using a microtome (Leica EM UC6). These sections were double-stained with uranyl acetate and lead citrate and subsequently examined at room temperature using a 120 kV transmission electron microscope (Tecnai Spirit, FEI). Images were captured with a CCD camera (MoradaG3, EMSIS).

### Quantitative reverse transcription PCR

Total RNA isolation from cells was performed using TRNzol Universal reagent (TIANGEN Biotech, Cat#DP424). Subsequent cDNA synthesis employed the FastKing RT Kit (TIANGEN Biotech, Cat#KR116). Quantitative reverse transcription PCR was conducted with SuperReal PreMix Plus (TIANGEN Biotech, Cat#FP205-03). The following primers were used:

β-actin-F, 5′-TACCACCATGTACCCAGGCA-3′, β-actin-R, 5′-CTCAGGAGGAGCAATGATCTTGAT-3′, CD63-F, 5′-CACACGGGAGAAAGGCCCAA-3′, CD63-R, 5′-ACAACCTGAACCGCTACACC-3′, LAMP1-F, 5′-CAGCACTCTTTGAGGTGAAAAAC-3′, LAMP1-R, 5′-ACGATCTGAGAACCATTCGCA-3′, LAMP2-F, 5′-TGTATTTGGCTAATGGCTCAGC-3′,LAMP2-R, 5′-TATGGGCACAAGGAAGTTGTC-3′, ATP6V1A-F, 5′-GACTAGCTAGGACTGGCCC-3′, ATP6V1A-R, 5′-CATTGTGTTAAATTTACCTGTCGC-3′, ATP6V0E1-F, 5′-GCATACCACGGCCTTACTGT-3′, ATP6V0E1-R, 5′-TGATAACTCCCCGGTTAGGAC-3′, ATP6V0D1-F, 5′-GCTACTTGGAGGGATTAGTGCG-3′, ATP6V0D1-R, 5′-GCGGAACTCTACTACCATCTTCT-3′, TFEB-F, 5′-GCTCCAACCCCGAGAAAGAG-3′, TFEB-R, 5′-CAGCGTGTTAGGCATCTGC-3′, CTSB-F, 5′-TCCTTGATCCTTCTTTCTTGCC-3′, CTSB-R, 5′-ACAGTGCCACACAGCTTCTTC-3′, CTSD-F, 5′-CCTGGCTTCGTCCTCCTTC-3′, CTSD-R, 5′-GGCGATGACTGCATGGAGT-3′, CTSF-F, 5′-CCCTGGAAGCCACACTAGAG-3′, and CTSF-R, 5′-GGGCTACAGTCCCTCCTCAG-3′.

### Western blotting

Cells and EVs were washed with PBS, and then lysed using radio immunoprecipitation assay lysis buffer (Beyotime, Cat#P0013B), with the addition of protease inhibitors (Merck, Cat#539137) and phosphatase inhibitors (Merck, cat#524627) for 30 min on ice. Subsequently, the samples were denatured in Laemmli buffer (containing 2% SDS, 5% 2-mercaptoethanol, 125 mM Tris–HCl, 10% glycerol, and 0.001% bromophenol blue, pH 6.8) and boiled at 100 °C for 10 min. Then, the samples were separated by SDS-PAGE and transferred to a polyvinylidene difluoride membrane (0.45 μm, Merck Millipore, cat#IPVH00010). The membranes were blocked in Phosphate Buffered Saline with Tween 20 containing 5% fat-free milk for 30 min and then incubated with primary antibodies overnight at 4 °C. Afterward, the membranes were washed three times with Phosphate Buffered Saline with Tween 20 and incubated with horseradish peroxidase–conjugated secondary antibodies at room temperature for 1 h. The blots were visualized using a ChemiScope imaging system (Clinx) with the enhanced chemiluminescence reagent (LabLead, cat#E1050). The band intensity was quantified by ImageJ (https://imagej.net/ij/download.html). The following commercial antibodies were used: anti-PERK antibody (Cell Signaling Technology, Cat#3192S), anti-ATF4 antibody (Cell Signaling Technology, Cat#11815), anti-XBP1s antibody (Cell Signaling Technology, Cat#12782S), anti-LAMP1 antibody (Abcam, Cat#ab208943), anti-CTSB antibody (ABclonal, Cat#A0967), anti-IRE1α antibody (Cell Signaling Technology, Cat#3294S), anti-CD63 antibody (Abcam, Cat#ab68418), anti-TSG101 antibody (Abcam, Cat#ab125011), anti-HRS antibody (Cell Signaling Technology, Cat#15087S), anti-ALIX antibody (Cell Signaling Technology, Cat#2171S), anti-GAPDH antibody (Gene-Protein Link, Cat#P01L01), anti-β-tubulin antibody (Gene-Protein Link, Cat#P01L06), anti-Mouse IgG-HRP (Jackson, Cat#115-035-003), and anti-Rabbit IgG-HRP (Jackson, Cat#111-035-003).

### Immunofluorescence staining

Cells were plated on coverslips and subjected to the treatments as indicated. After removing the culture medium, cells were washed with PBS and fixed with 4% paraformaldehyde for 15 min. Subsequently, cells were permeabilized with 0.1% Triton X-100 for 10 min. Blocking was performed with 1% BSA for 30 min, followed by overnight incubation with primary antibodies at 4 °C. Then, the cells were washed three times with PBS, incubating with secondary antibodies at room temperature for 1 h. Following PBS washing, the cells were incubated at room temperature with Hoechst 33342 for 10 min. For MVB number analysis, imaging was carried out using FV3000 (Olympus) using 60 × /1.4 oil-immersion objective, and the images were analyzed with ImageJ software. For MVB and lysosome colocalization analysis, imaging was carried out using LSM980 (Zeiss) equipped with a 63 × /1.4 oil-immersion objective, and the images were analyzed with Imaris 10.0 (https://imaris.oxinst.com/). For three-dimensional imaging, the images were taken by LSM980 using 63 × /1.4 oil-immersion objective. Three-dimensional reconstruction, surface rendering, and colocalization analysis were performed by Imaris 10.0. The following commercial antibodies were used: anti-CD63 antibody (MBL, Cat#D263-3), anti-LAMP1 antibody (Abcam, Cat#ab237307), anti-Flag antibody (Proteintech, Cat#66008-3-Ig), anti-EEA1 antibody (Cell Signaling Technology, Cat#C45B10), anti-Rabbit Alexa Fluor 555 (BioLegend, Cat#406412), and anti-Rat Alexa Fluor 488 (BioLegend, Cat#405418).

### Magic Red assay

Magic Red (Bio-Rad, Cat#ICT937) was used to analyze the CTSB enzymatic activity in lysosomes as an indicator of lysosome function. Briefly, the cells were seeded in confocal plates. The Magic Red solution was prepared by dissolving the stock reagent in 50 μl dimethyl sulfoxide, followed by 1:10 dilution in deionized water. The cells were treated with Magic Red solution at 37 °C for 15 min (20 μl of the optimized Magic Red solution was added to each 500 μl culture medium). Subsequently, the cells were washed with PBS and fixed with 4% paraformaldehyde for 15 min. Then, nuclei were stained with Hoechst 33342 for 10 min. Fluorescence imaging was subsequently conducted using FV3000 (Olympus). ImageJ software was used to analyze fluorescence intensity per cell.

### LysoSensor assay

The LysoSensor Green DND-189 (Thermo Fisher Scientific, Cat#L7535) was used to analyze the lysosome acidity. Briefly, the cells were seeded in 6-well culture plates. Upon completion of cellular treatments, the cells were treated with 1 μM LysoSensor fluorescent probe dyes at 37 °C for 15 min. Subsequent to staining, residual dye solution was carefully aspirated, and the cells underwent two gentle washes with PBS. Cellular dissociation was achieved through trypsinization. The resulting cell suspension was centrifuged at 1000*g* for 3 min. Following a final PBS wash, cells were resuspended in 500 μl PBS and transferred to sterile flow cytometry tubes. Quantitative fluorescence intensity analysis was performed using a flow cytometer FACSCalibur (BD Biosciences) with 488 nm excitation.

### Mass spectrometry

To analyze the proteins in EVs collected from WT, IRE1α KO and PERK KO B16.F10 cells, EVs were enriched as described previously in the methods section. Then, 100 μg of EV proteins was subjected to label-free proteomics mass spectrometry using the nanoLC-Orbitrap Eclipse (Thermo Fisher Scientific). For WT-EVs, IRE1α KO-EVs, and PERK KO-EVs analysis, KEGG enrichment analysis with the Kyoto Encyclopedia of Genes and Genomes (KEGG) database was accomplished by https://www.bioinformatics.com.cn, an online platform for data analysis and visualization (55).

### Generation of PERK and ATF4 KO cell lines

PERK and ATF4 KO cell lines were created using the CRISPR/Cas9 system. Briefly, guide RNA sequences specific to mouse PERK (5′- CCAAACACTGTCTCCGACG-3′), ATF4 (5′-GATGTCCCCCTTCGACCAGT-3′), and IRE1α (5′-AGAGGACGGGCTCCATCAAG-3′) were acquired through the CRISPR design tool (http://crispor.tefor.net/). B16.F10 cells were transiently transfected with the pX458-hpCas9-EGFP vector containing the sgRNA sequences. After 24 h of incubation, cells were digested with trypsin, and single cells expressing EGFP were sorted into a 96-well plate containing complete DMEM using flow cytometry after passing through a 70 μm cell strainer. Positive clones were identified by Western blot or DNA sequencing to confirm successful knockout.

### Generation of CD63 and CTSB knockdown cell lines

The lentiviral system was implemented to generate knockdown cell lines. Briefly, 293T cells were cotransfected with three plasmid constructs: psPAX2, VSV-G, and pLKO.1_shCD63/shCTSB or the nontargeting control pLKO.1_shNT. The specific shRNA oligonucleotides employed were designed as follows:

shCD63-F, 5′-CCGGCCTAAGGTTAAGTCGCCCTCGCTCGAGCGAGGGCGACTTAACCTTAGGTTTTTG-3′, shCD63-R, 5′-AATTCAAAAACCTAAGGTTAAGTCGCCCTCGCTCGAGCGAGGGCGACTTAACCTTAGG-3′, shCTSB-F, 5′-CCGGCCTGCTTACTTGCTGTGGTATCTCGAG ATACCACAGCAAGTAAGCAGGTTTTTG-3′, shCTSB-R, 5′-AATTCAAAAA CCTGCTTACTTGCTGTGGTATCTCGAGATACCACAGCAAGTAAGCAGG-3′, shNT-F, 5′-CCGGCCTAAGGTTAAGTCGCCCTCGCTCGAGCGAGGGCGACTTAACCTTAGGTTTTTG-3′, and shNT-R, 5′-AATTCAAAAACCTAAGGTTAAGTCGCCCTCGCTCGAGCGAGGGCGACTTAACCTTAGG-3′.

### Generation of stable cell lines

Construction of stably transfected cell lines using lentiviral system. Briefly, for establishing T-REx-PERK cell lines for doxycycline-inducible expression of PERK, 293T cells were cotransfected with three plasmid constructs: psPAX2, VSV-G, and pCW-PERK. For establishing stably expression of ATF4 in ATF4 KO B16.F10 cells, 293T cells were cotransfected with three plasmid constructs: psPAX2, VSV-G and lentiCRISPR V2-ATF4. The lentivirus was collected at 48 h and filtered through a 0.22 μm pore polyethersulfone filter. Subsequently, the lentivirus was used to infect PERK KO or ATF4 KO B16.F10 cells with 7.5 ug/ml polybrene (YEASEN, Cat#40804ES76) for 24 h. Finally, 5 ug/ml puromycin (lnvivoGen, Cat#ant-pr-1) was used to screen cells that were successfully transduced with the puromycin resistance gene.

### Data analysis

Statistical analyses were performed using GraphPad Prism 9 software (http://www.graphpad.com). The statistical analyses are described in detail in figure legends. All data were shown as the mean ± SD. In all figures: ∗*p* < 0.05, ∗∗*p* < 0.01, and ∗∗∗*p* < 0.001. Significance was concluded at *p* < 0.05.

## Data availability

All data are contained within the article or will be made available upon written request to the corresponding author.

## Supporting information

This article contains supporting information.

## Conflict of interest

The authors declare that they have no conflicts of interest with the contents of this article.
