# Supplementary material for: PERK and IRE1α promote exosome secretion via blocking lysosomal degradation of multiple vesicular body
Source: J Biol Chem. 2025 Jul 21;301(8):110504. doi: 10.1016/j.jbc.2025.110504 (PMC12363578; doi:10.1016/j.jbc.2025.110504)
Supplement: Supplementary Data [file mmc1.docx]

**Supporting Information for**

**PERK and IRE1α promote exosome secretion via blocking lysosomal degradation of multiple vesicular body**

Shixin Zhou^1,2^, Zihan Zhou^1,2^, Si Chen^1,2^, Ming Wang^1,2^, Likun Wang^1,2,*^

Likun Wang

Email: wanglikun@ibp.ac.cn

**This PDF file includes:**

Figures S1 to S5

Legends for Videos S1 to S5

**Figure S1.** **IRE1α deletion but not PERK deletion reduces EVs secretion.** *A,* western blot analysis of IREα in WT and IREα KO B16.F10 cells treated with 10 nM Tg for 24 h. *B,* left, western blot analysis of EVs purified from cell culture supernatants from equal numbers of WT and IREα KO B16.F10 cells treated as in (*A*). Right, quantiﬁcation of band intensities of EVs marker proteins in EVs enriched from three independent experiments. *C,* quantification of EVs concentration by NTA (n = 3), normalized to cell number. EVs were obtained as in (*B*). *D,* western blot analysis of UPR marker proteins and EVs marker proteins in WT and PERK KO B16.F10 cells treated with DMSO or 10 nM Tg for 24 h. *E,* left, western blot analysis of EVs purified from cell culture supernatants from equal numbers of WT and PERK KO B16.F10 cells treated as in (*D*). Right, quantiﬁcation of band intensities of EVs marker proteins in EVs enriched from three independent experiments. *F,* quantification of EVs concentration by NTA (n = 3), normalized to cell number. EVs were obtained as in (*E*). *G,* NTA analysis of EVs enriched in the culture medium of WT and PERK KO B16.F10 cells treated with 10 nM Tg and 500 nM KIRA8 for 24 h. Statistical analysis was calculated by one-way ANOVA. Data were shown as mean ± SD. **p* < 0.05, ***p* < 0.01 and ****p* < 0.001.

**Figure S2. Isolated EVs are derived from the endocytosis pathway.** *A,* Venn diagram analysis of protein differences in EVs derived from WT, IRE1α KO, and PERK KO B16.F10 cells. EVs were extracted from the culture medium after treating cells with 10 nM Tg for 24 h, followed by mass spectrometry analysis. *B,* KEGG pathway analysis of the common proteins in EVs derived from WT, IRE1α KO, and PERK KO B16.F10 cells treated as in (*A*).

**Figure S3. Knocking down CD63 does not interfere with EVs secretion in IRE1α-inhibited cells.** *A* and *B,* detection of the CD63-knockdown efficiency by qPCR (*A*) and western blot (*B*) in B16.F10 cells. *C,* left, western blot analysis of EVs derived from equal numbers of shNT and shCD63 B16.F10 cells treated with 10 nM Tg and 500 nM KIRA8 for 24 h. Right, quantiﬁcation of band intensities of EVs marker proteins in EVs enriched from three independent experiments. *D,* quantification of EVs concentration by NTA (n = 3), normalized to cell number. EVs were obtained as in (*C*). Statistical analysis was calculated by one-way ANOVA. Data were shown as mean ± SD. ns, not significant; ****p* < 0.001.

**Figure S4. PERK deletion has no effect on MVB numbers, MVB-lysosome fusion, or lysosome acidity in IRE1α-competent cells.** *A,* electron microscopy images of MVBs (red arrows) and ILVs (blue arrows) in WT and PERK KO B16.F10 cells treated with DMSO or 10 nM Tg for 6 h. Scale bar, 200 nm. *B,* left, quantiﬁcation of the number of MVBs per cell per 100 μm^2^ cytoplasm. Each dot represents the number of MVBs per section, n = 20. Right, quantiﬁcation of the number of ILVs per MVB, n = 20. *C,* quantification of the percentage of CD63 and LAMP1 co-localized volume among the total CD63 volume per cell with three-dimensional imaging in WT and PERK KO B16.F10 cells treated with 10 nM Tg for 6 h, n = 5. *D,* flow cytometric analysis of LysoSensor mean fluorescence intensity (MFI) in WT and PERK KO B16.F10 cells treated with 10 nM Tg for 6 h to evaluate lysosomal acidity. The left panel shows a representative result, and the right panel presents statistical data from three independent experiments. Statistical analysis was calculated by unpaired t test analysis, two tailed (*C* and *D*) or one-way ANOVA (*B*). Data were shown as mean ± SD. ns, not significant; ***p* < 0.01.

**Figure S5. PERK promotes EVs secretion through ATF4.** *A,* western blot analysis of UPR marker proteins and EVs marker proteins in WT B16.F10 cells treated with 500 nM KIRA8, as well as in WT, PERK KO and ATF4-rescued PERK KO B16.F10 cells treated with 10 nM Tg and 500 nM KIRA8 for 6 h. *B,* left, western blot analysis of EVs purified from cell culture supernatants from equal numbers of WT, PERK KO and ATF4-rescued PERK KO B16.F10 cells treated with 10 nM Tg and 500 nM KIRA8 for 24 h. EVs were blotted for EVs marker proteins HRS, ALIX, CD63 and TSG101. Right, quantiﬁcation of EVs marker protein levels in the EVs obtained from cell culture supernatants of WT, PERK KO and ATF4-rescued PERK KO B16.F10 cells treated with 10 nM Tg and 500 nM KIRA8 for 24 h in three independent experiments. *C,* quantification of EVs concentration by NTA (n = 3), normalized to cell number. EVs were obtained as in (*B*). *D,* qPCR analysis of lysosome biogenesis-related genes in WT, ATF4 KO and ATF4-rescued ATF4 KO B16.F10 cells treated with 10 nM Tg and 500 nM KIRA8 for 6 h (data from three independent experiments). Statistical analysis was calculated by one-way ANOVA. Data were shown as mean ± SD. ns, not significant; **p* < 0.05, ***p* < 0.01 and ****p* < 0.001.

Videos S1-S2. Three-dimensional imaging analysis of CD63 and LAMP1 co-localization in WT (Video S1), and PERK KO (Video S2) B16.F10 cells treated with 10 nM Tg for 6 h. Cyan spheres indicate MVB, and blue spheres indicate lysosome.

Videos S3-S5. Three-dimensional imaging analysis of CD63 and LAMP1 co-localization in WT (Video S3), T-REx-PERK (endogenous PERK KO background) (Video S4), and Dox-pretreated T-REx-PERK (endogenous PERK KO background) (Video S5) B16.F10 cells treated with 10 nM Tg and 500 nM KIRA8 for 6 h. Cyan spheres indicate MVB, and blue spheres indicate lysosome.
